# 833. Interaction between *Aspergillus fumigatus* and non-tuberculous mycobacteria

**DOI:** 10.1093/ofid/ofad500.878

**Published:** 2023-11-27

**Authors:** Hotaka Namie, Takahiro Takazono, Satoru Koga, Masato Tashiro, Hiroshi Mukae, Koichi Izumikawa

**Affiliations:** Nagasaki University Graduate School of Biomedical Sciences, Nagasaki, Nagasaki, Japan; Nagasaki University Graduate School of Biomedical Sciences, Nagasaki, Nagasaki, Japan; Nagasaki University, Nagasaki, Nagasaki, Japan; Nagasaki University Graduate School of Biomedical Sciences, Nagasaki, Nagasaki, Japan; Nagasaki University, Nagasaki, Nagasaki, Japan; Nagasaki University, Nagasaki, Nagasaki, Japan

## Abstract

**Background:**

Patients with pulmonary non-tuberculous mycobacteriosis (NTM) have been increasing in Japan in recent years. Furthermore, NTM cases complicated with pulmonary aspergillosis are associated with a poor prognosis. However, little is known about the interaction between *Aspergillus spp.* and non-tuberculous mycobacterium.

**Methods:**

To investigate the effect of NTM metabolites on *A. fumigatus* growth and gene expression, supernatants of *M. avium* (ATCC 700737) and *M. abscessus* (ATCC 19977) were added to *A. fumigatus* (Figure 1 and 2).

**Figure:**
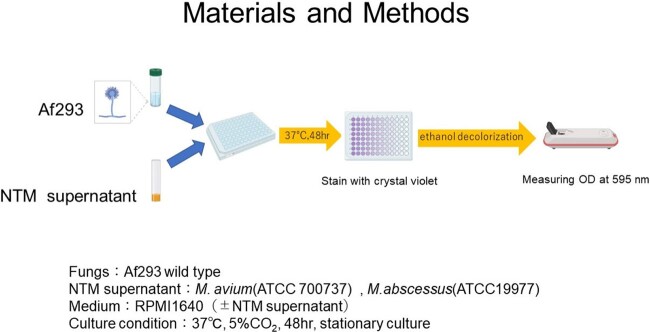

**Figure:**
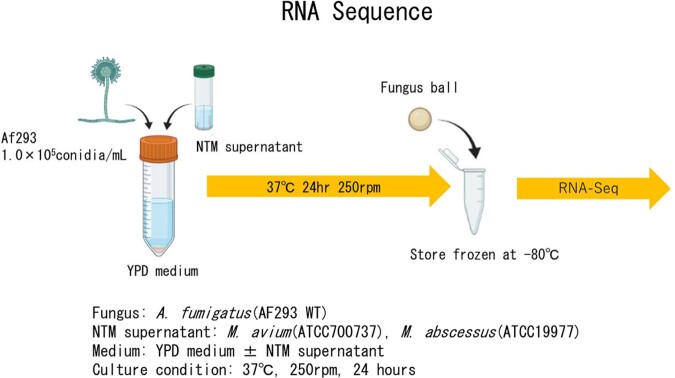

**Results:**

The biofilms and biomass of *A. fumigatus* exposed to culture supernatant of each NTM were significantly increased compared to the control (Figure 3). The expression of genes involved in the synthesis of secondary metabolites of *A. fumigatus*, such as gliotoxin, was decreased after the exposure to supernatant by RNA sequencing analysis (Table 1,2).

**Figure:**
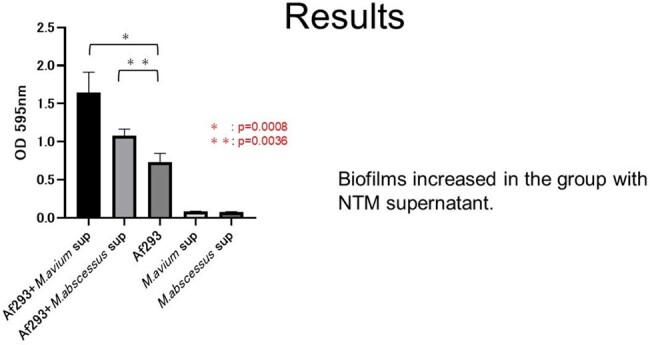

**Figure:**
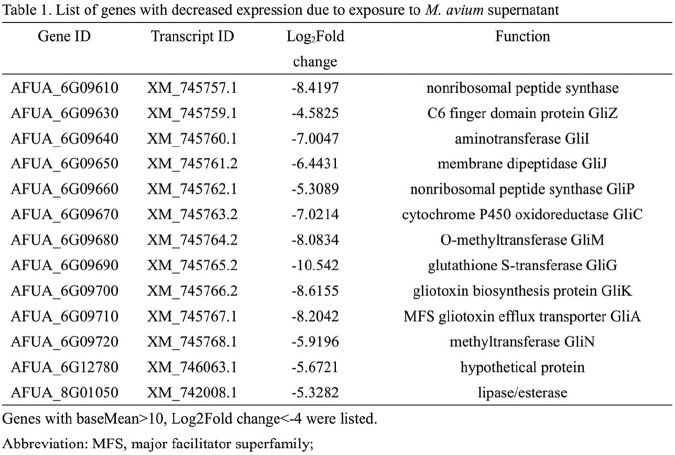

**Figure:**
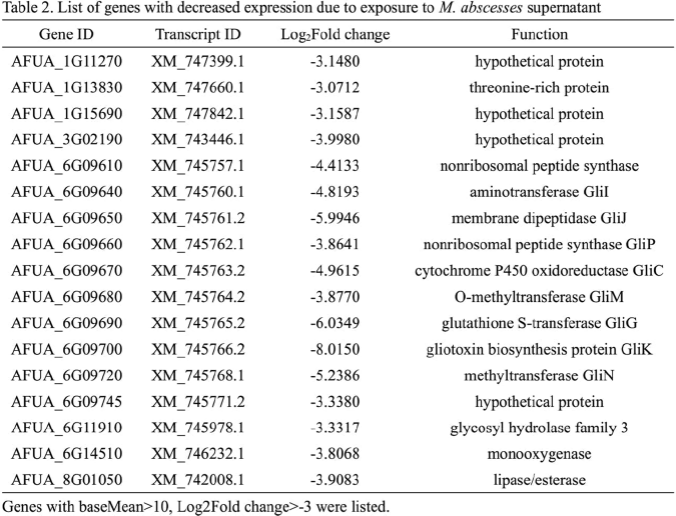

**Conclusion:**

Our results suggest that exposure to culture supernatant of NTM may support the growth of *A. fumigatus* while suppressing the production of some secondary metabolites. We are now trying to reproduce this phenotype in mouse model and to identify the causative agent from NTM culture supernatant and elucidate the mechanism further.

**Disclosures:**

**Masato Tashiro, MD, PhD**, Asahi Kasei Pharma Corporation: Advisor/Consultant|Asahi Kasei Pharma Corporation: Honoraria|Sumitomo Pharma Co., Ltd.: Honoraria **Koichi Izumikawa, M.D., Ph.D.**, Asahi Kasei Pharma Corporation: Grant/Research Support|Asahi Kasei Pharma Corporation: Honoraria|Astellas Pharma Inc.: Honoraria|DAIICHI SANKYO COMPANY, LIMITED: Grant/Research Support|DAIICHI SANKYO COMPANY, LIMITED: Honoraria|KYORIN Pharmaceutical Co., Ltd.: Honoraria|Merck & Co., Inc.: Honoraria|Pfizer Japan Inc.: Honoraria|Shionogi & Co., Ltd.: Grant/Research Support|Shionogi & Co., Ltd.: Honoraria|Sumitomo Pharma Co., Ltd.: Grant/Research Support|Sumitomo Pharma Co., Ltd.: Honoraria|TAIHO PHARMACEUTICAL CO., LTD.: Grant/Research Support

